# Supplementary material for: Transition metal-doped Ni-rich layered cathode materials for durable Li-ion batteries
Source: Nat Commun. 2021 Nov 12;12:6552. doi: 10.1038/s41467-021-26815-6 (PMC8589951; doi:10.1038/s41467-021-26815-6)
Supplement: Supplementary file 1 — Supplementary information [file 41467_2021_26815_MOESM1_ESM.docx]

**Supplementary Information**

Transition metal-doped Ni-rich layered cathode materials for durable Li-ion batteries

H. Hohyun Sun^1,6^, Un-Hyuck Kim^2,6^, Jeong-Hyeon Park^3^, Sang-Wook Park^4^, Dong-Hwa Seo^4^, Adam Heller^1^, C. Buddie Mullins^1,5,^*, Chong S. Yoon^3^, Yang-Kook Sun^2,^*

^1^McKetta Department of Chemical Engineering, The University of Texas at Austin, Austin, Texas, 78712-1589, United States

^2^Department of Energy Engineering, Hanyang University, Seoul 04763, Republic of Korea
^3^Department of Materials Science Engineering, Hanyang University, Seoul 04763, Republic of Korea

^4^School of Energy and Chemical Engineering, Ulsan National Institute of Science and Technology (UNIST), Ulsan 44919, Republic of Korea

^5^Department of Chemistry, The University of Texas at Austin, Austin, Texas, 78712-1589, United States
^6^These authors contributed equally: H. Hohyun Sun, Un-Hyuck Kim.

Corresponding author: [mullins@che.utexas.edu](mailto:mullins@che.utexas.edu), [yksun@hanyang.ac.kr](mailto:yksun@hanyang.ac.kr).


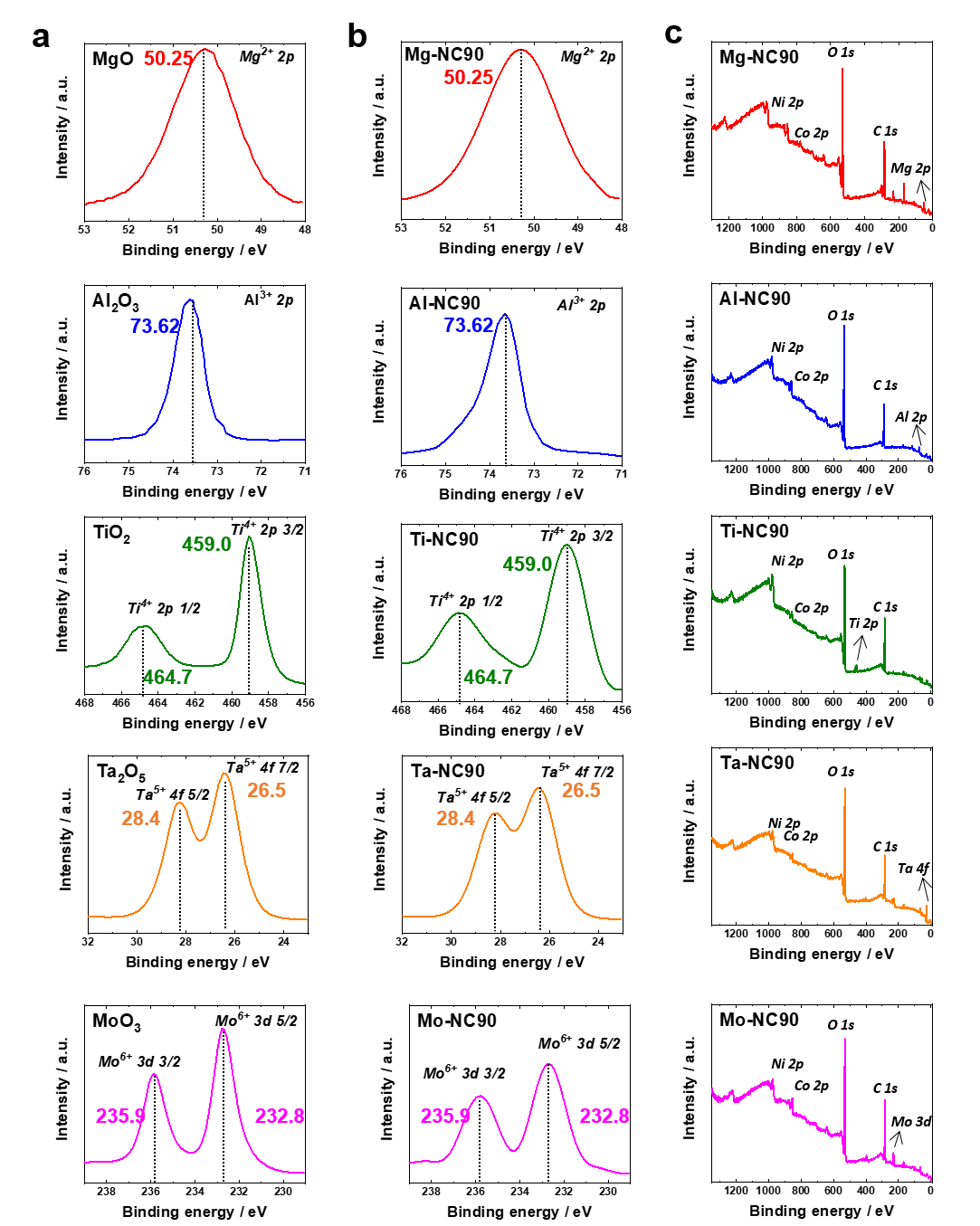


**Supplementary Figure 1:** X-ray photoelectron spectroscopy (XPS) results of (a) the dopant parent materials, MgO, Al_2_O_3_, TiO_2_, Ta_2_O_5_, and MoO_3_, (b) corresponding doped cathode materials, Mg-NC90, Al-NC90, Ti-NC90, Ta-NC90, and Mo-NC90, and (c) surevey spectra of the doped cathode materials.

**Supplementary Figure 2:** Coulombic efficiencies of NC90, Mg-NC90, Al-NC90, Ti-NC90, Ta-NC90, and Mo-NC90 cathodes in half cells at (a) 30 °C and (b) at 60 ° C.


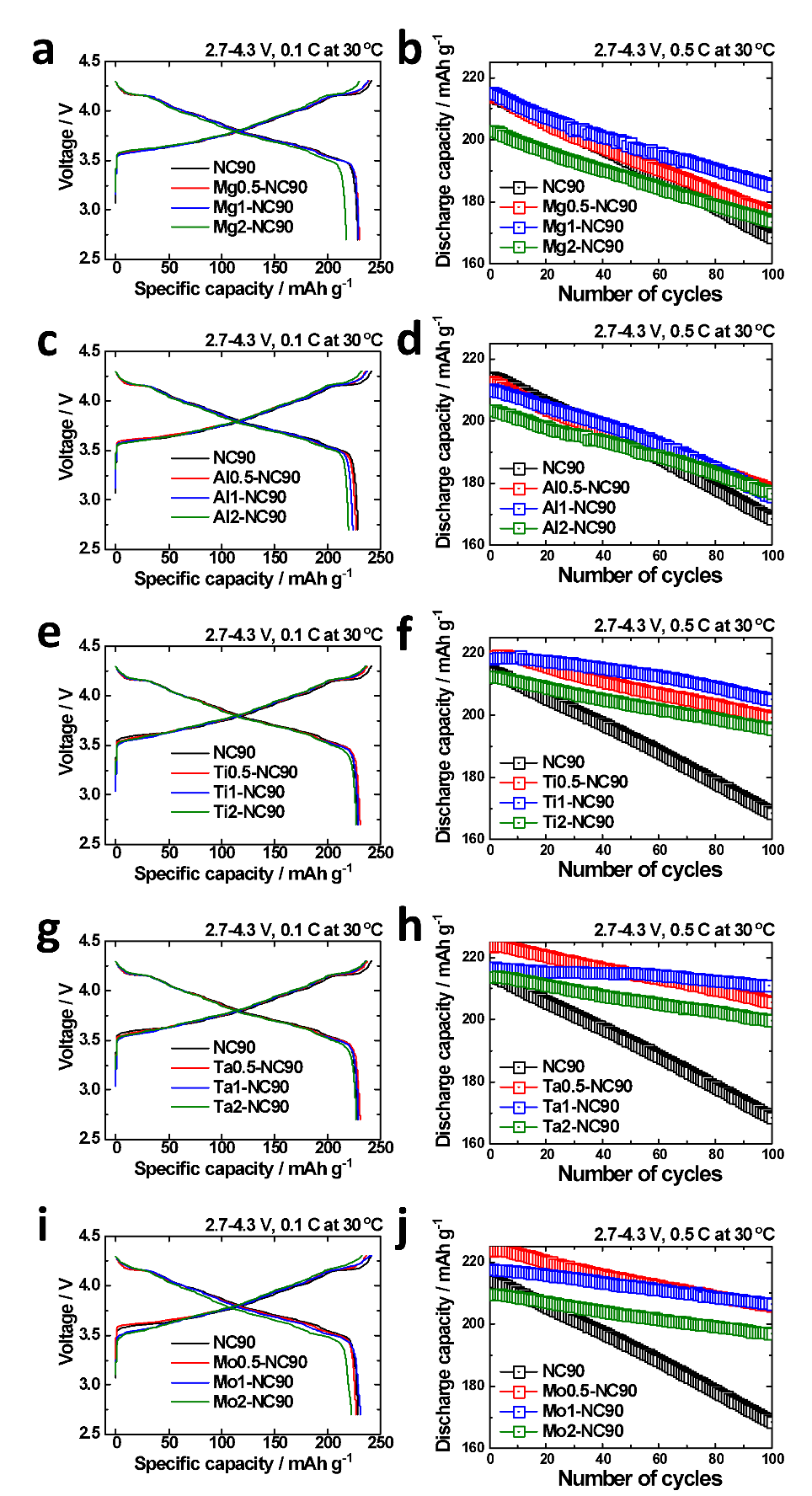


**Supplementary Figure 3:** Half-cell cycling of differing dopant concentrations (0.5, 1, and 2 mol %) of Mg^2+^, Al^3+^, Ti^4+^, Ta^5+^, and Mo^6+^: (a, c, e, g, i) first charge-discharge cycle curves at 0.1 C = 18 mA g^-1^ and (b, d, f, h, j) cycling at 0.5 C = 90 mA g^-1^ over 100 cycles at 30 °C and between 2.7 – 4.3 V voltage window.

**
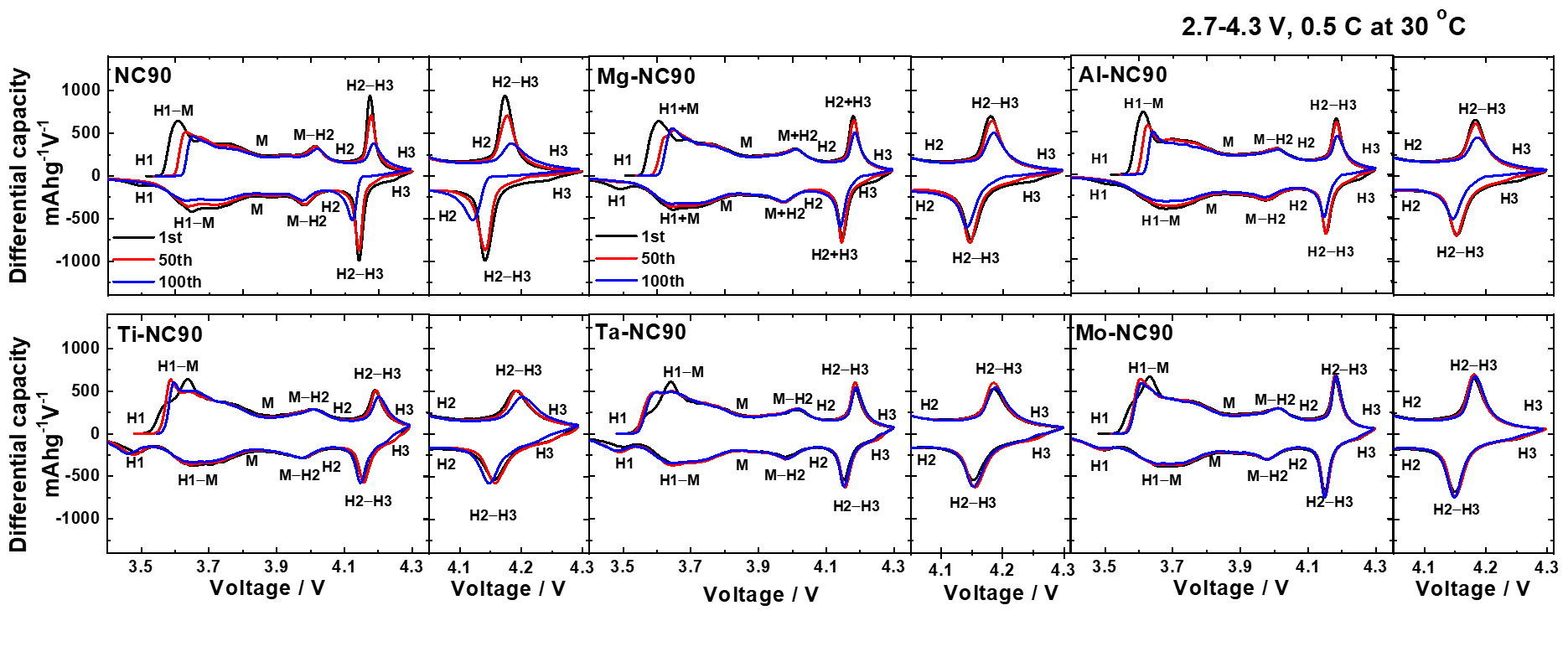
**

**Supplementary Figure 4:** The 1^st^, 50^th^, and 100^th^ cycle *dQ dV^−1^* curves associated with NC90, Mg-NC90, Al-NC90, Ti-NC90, Ta-NC90, and Mo-NC90 cathodes, corresponding to the cycling performances in Figure 1b. Note the reversible H2–H3 phase transitions of the Ta-NC90 and Mo-NC90 cathodes.


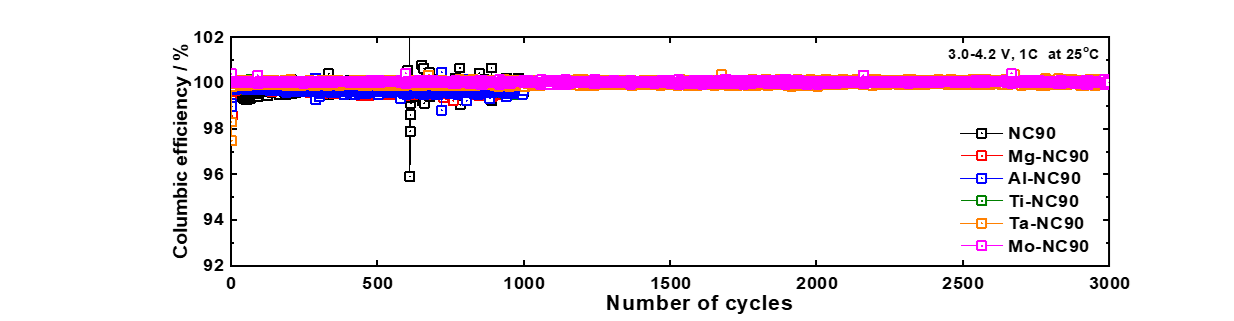


**Supplementary Figure 5:** Coulombic efficiencies of NC90, Mg-NC90, Al-NC90, Ti-NC90, Ta-NC90, and Mo-NC90 cathodes in in pouch-type full cells at 25 °C.


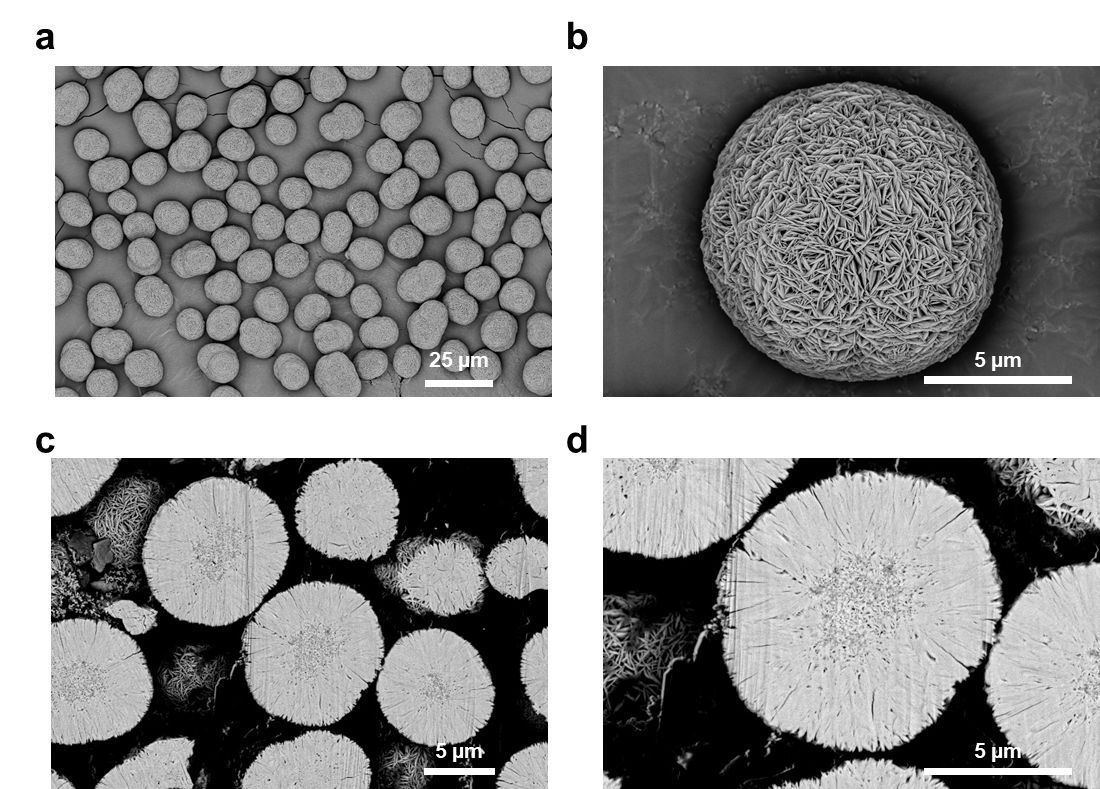


**Supplementary Figure 6:** SEM images of the (a, b) surface and (c, d) cross-sections of the precursor, [Ni_0.91_Co_0.09_](OH)_2_. The particles comprise thin, needle-like grains.


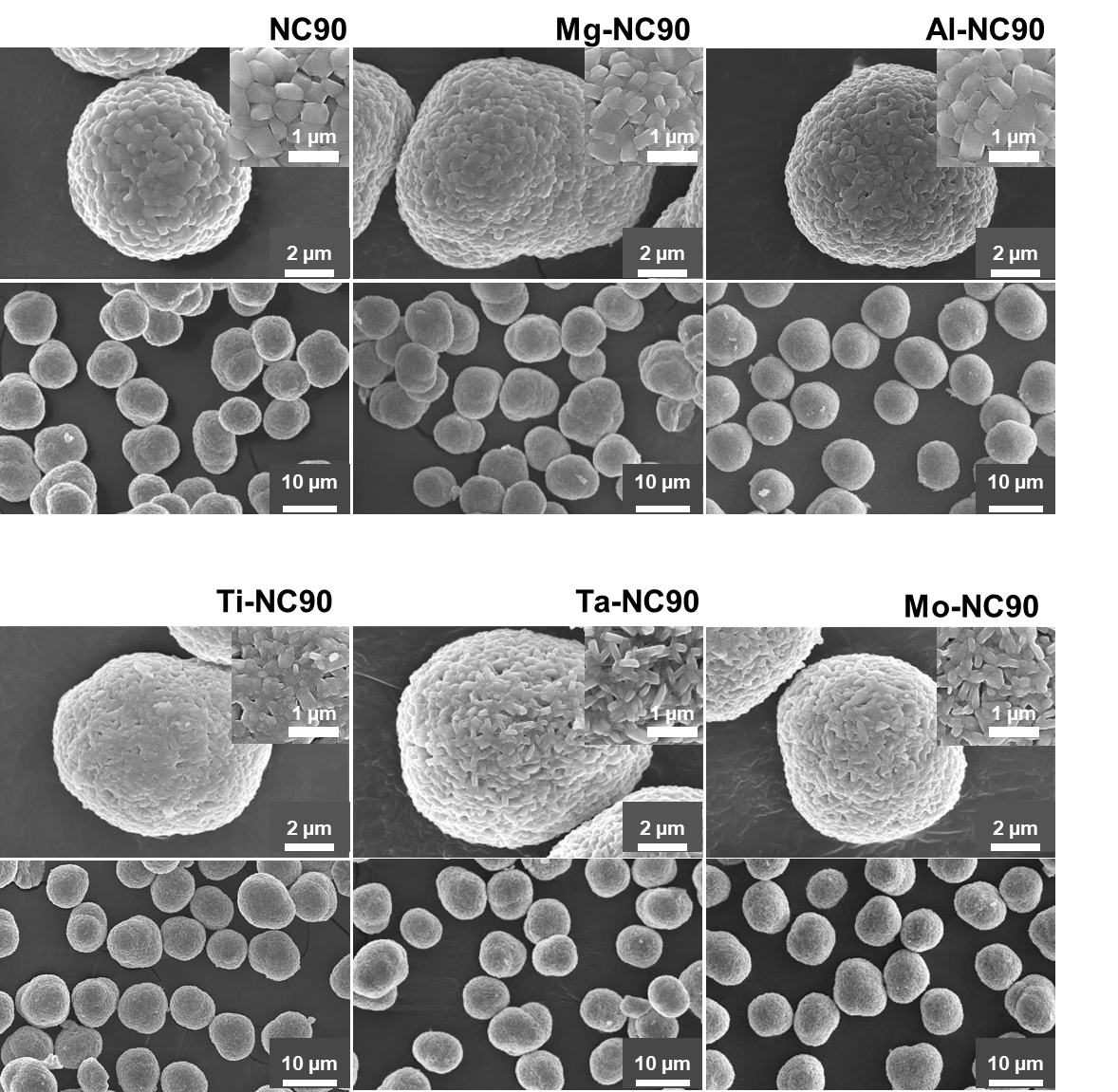


**Supplementary Figure 7:** SEM images of the as-synthesised NC90, Mg-NC90, Al-NC90, Ti-NC90, Ta-NC90, and Mo-NC90 cathode particles. The surface morphologies of the particles appear similar.


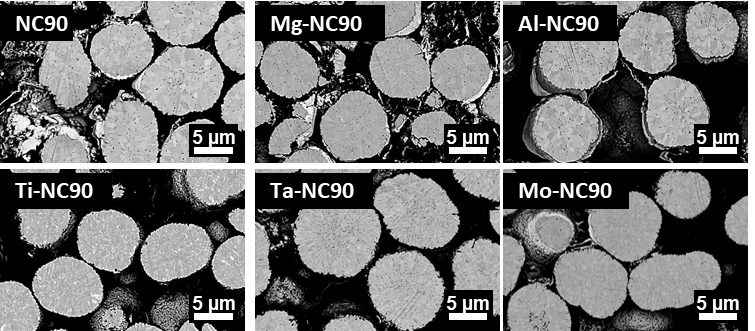


**Supplementary Figure 8:** Cross-sectional SEM images of the as-synthesised NC90, Mg-NC90, Al-NC90, Ti-NC90, Ta-NC90, and Mo-NC90 cathode particles. The SEM images show that the grain characteristics of each cathode are consistent.

**Supplementary Figure 9:** TEM images with corresponding SAED patterns of the primary particles of as-prepared (a) Al-NC90 (equiaxed primary particle) and (b) Ta-NC90 (radially oriented particle) cathode materials that show the parallel alignment of their longitudinal axes to the *a*-axes. Model and method by which the angle between the *a*-axis of grain and the corresponding secondary-particle radial line were measured in the cases of (c) equiaxed and (d) radially oriented primary particles.


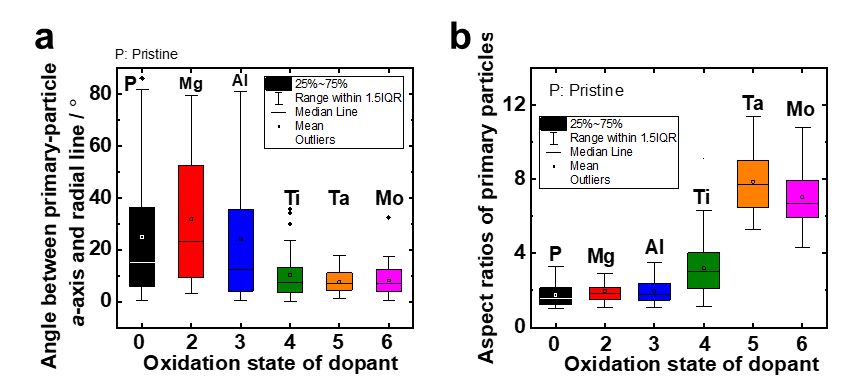


**Supplementary Figure 10:** Measured (a) relative primary-particle angles and (b) primary-particle aspect ratios of the cathode materials in Supplementary Figure 8. The error bars in the box chart represent the standard deviation calculated over 50 particles for each cathode.

**Supplementary Figure 11:** (a) Schematic illustrating the change in the angles between adjacent grains before and after cycling. (b) The angles between adjacent grains before and after 1000 cycles. The error bars in the box chart represent the standard deviation calculated over 20 particles for each cathode.


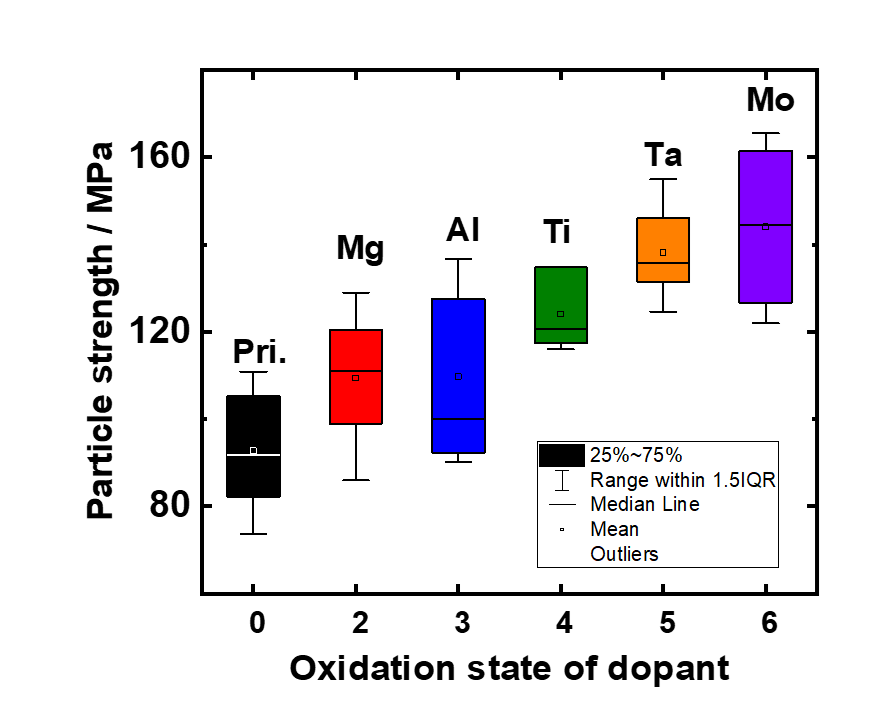


**Supplementary Figure 12:** Strengths (determined through micro-compression testing) of the cathode particles (measured seven times). The strength of the cathode particle increases with the increasing oxidation state of the dopant. The error bars in the box chart represent the standard deviation calculated over 7 particles for each cathode.

**Supplementary Figure 13:** (a) *R_ct_* values of NC90, Mg-NC90, Al-NC90, Ti-NC90, Ta-NC90, and Mo-NC90 cathodes as a function of cycle number. (b) The 1^st^, 25^th^, 50^th^, 75^th^, and 100^th^ Nyquist plots of the electrochemical impedance of the six cathodes.


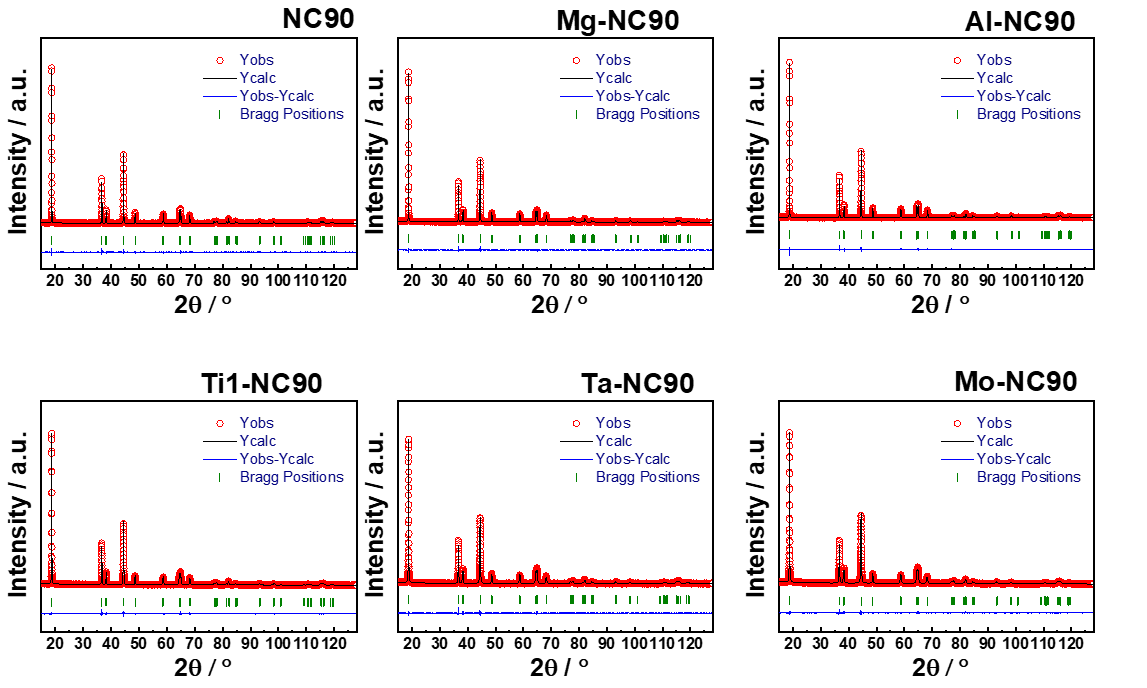


**Supplementary Figure 14:** Rietveld-refined XRD patterns of NC90, Mg-NC90, Al-NC90, Ti-NC90, Ta-NC90, and Mo-NC90 cathode materials.

**Supplementary Figure 15:** Hexagonal crystal structure characteristics of the as-synthesised cathode materials determined from their Rietveld-refined XRD patterns: (a) unit cell volume and (b) intensity ratio of the (003) and (104) peaks (expressed as (003)/(104)), which indicate the degree of Li/Ni cation mixing.

**Supplementary Figure 16:** In situ XRD determination of the change in the (a) *a-* and *c-*axis lattice parameters and (b) unit cell volume of the six cathodes during initial charge.


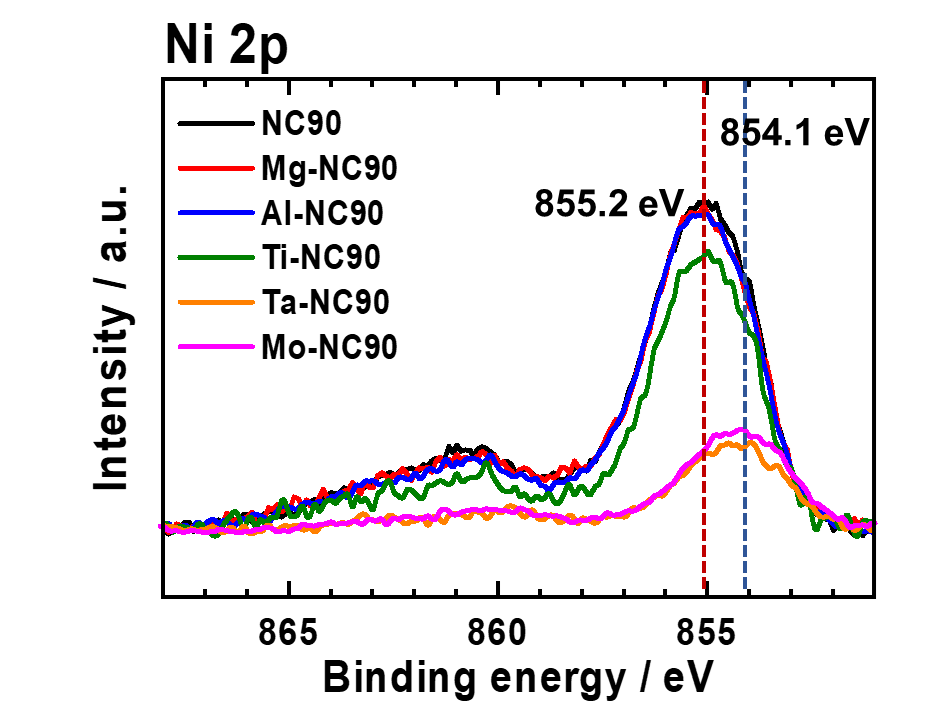


**Supplementary Figure 17:** XPS Ni *2p* spectra of the six cathode materials. In the XPS spectra of the Ta-NC90 and Mo-NC90 cathodes, the Ni *2p_3/2_* peak appears at lower binding energies, indicating the presence of significant amounts of Ni in an oxidation state of +2.

**Supplementary Figure 18:** HR-TEM images of (a) Ta-NC90 and (b) Al-NC90 cathodes in the deeply charged state of 4.5 V and corresponding SAED images. The SAED images show that the cation-ordered superlattice structure of Ta-NC90 is well maintained even at 4.5 V.


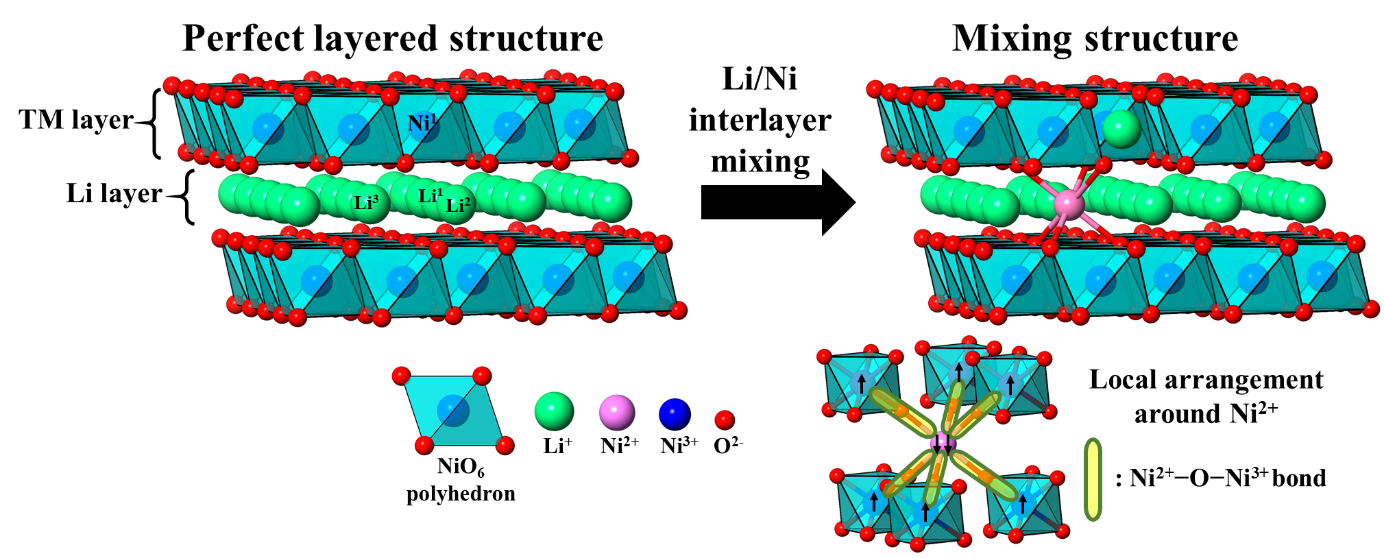


**Supplementary Figure 19:** Li/Ni interlayer mixing process in Li_32_Ni_32_O_64_. Three Li sites (Li1, Li2, and Li3) for Li/Ni interlayer mixing are considered; the exchange between Ni1 and Li3 sites is most stable. The linear Ni^2+^ (Li layer, down-spin)–O^2-^–Ni^3+^ (TM layer, up-spin) bonds introduced by Li/Ni interlayer mixing results in moderate super-exchange interactions via the σ bonds between the *e_g_* orbitals of Ni^2+^ in the Li layer and the *p* orbitals of O^2-^.

**Supplementary Figure 20:** Li/Ni interlayer mixing energy (E_mixing_) values of Li_32_Ni_31_MO_64_ (M = Mg^2+^, Al^3+^, Ti^4+^, Ta^5+^, and Mo^6+^) based on (a) P2_1_/c LiNiO_2_ structure and (b) C2/m LiNiO_2_ structure. To compare E_mixing_ systematically, we used the energy of Li_32_Ni_31_MgO_64_ with Mg substituted into the TM layer, which is less stable than the model with Mg substituted into the Li layer.


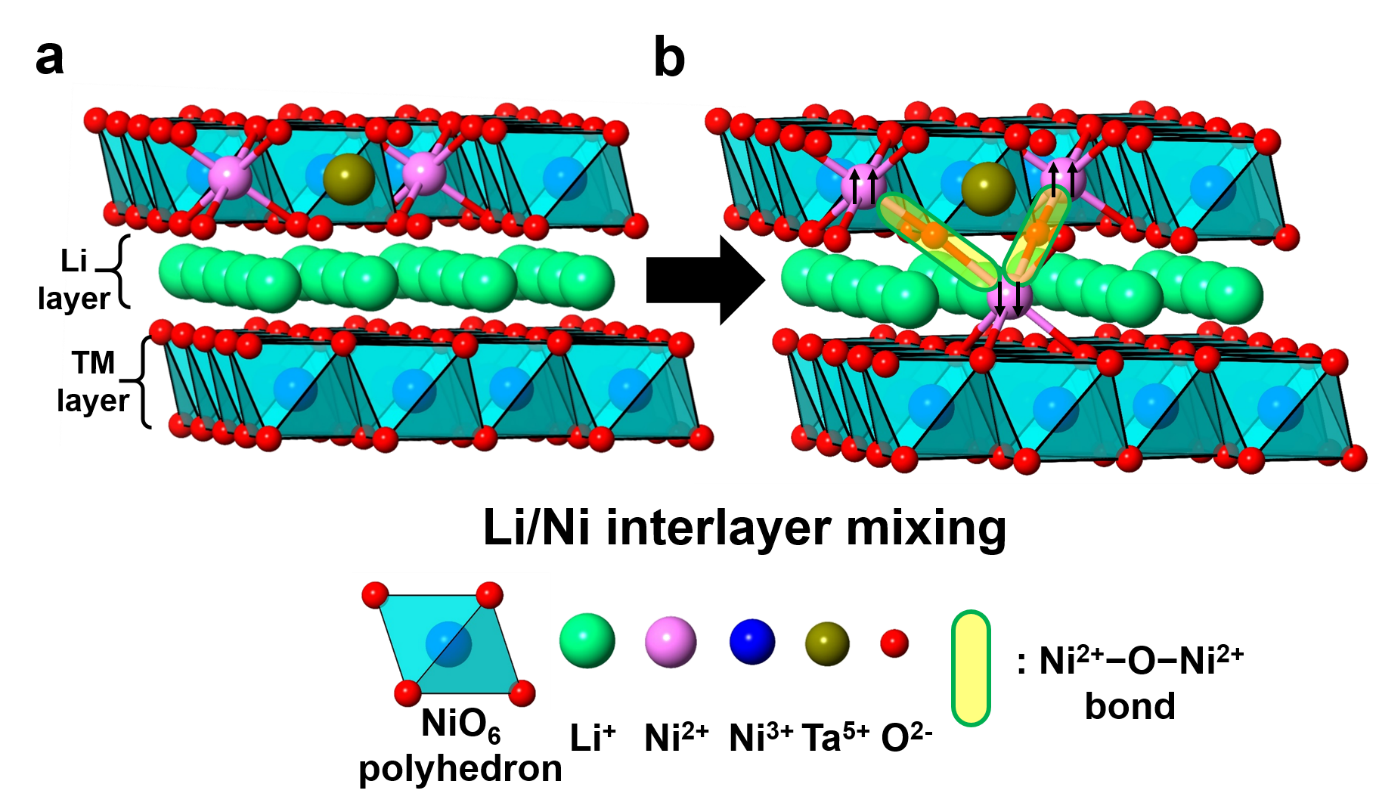


**Supplementary Figure 21:** (a) Li_32_Ni_31_TaO_64_ structure without Li/Ni interlayer mixing (b) Li_32_Ni_31_TaO_64_ structure with Li/Ni interlayer mixing. The linear Ni^2+^(Li layer, down-spin)−O^2-^−Ni^2+^(TM layer, up-spin) bonds introduced by Li/Ni interlayer mixing result in strong super-exchange interactions via the σ bonds between the *e_g_* orbitals of Ni^2+^ and the *p* orbitals of O^2-^. Note that Mg^2+^ and Al^3+^ do not reduce Ni^3+^ to Ni^2+^, so Li_32_Ni_31_MgO_64_ and Li_32_Ni_31_AlO_64_ do not possess strong super-exchange interaction. The difference between Mg^2+^ and Al^3+^ doped structures is the strain difference between the dopant and Ni^3+^. The larger E_mixing_ of Mg^2+^ is mainly attributed to the larger strain between Mg^2+^(0.72 Å) and Ni^3+^ (0.56 Å) than that of Al^3+^ (0.535 Å).


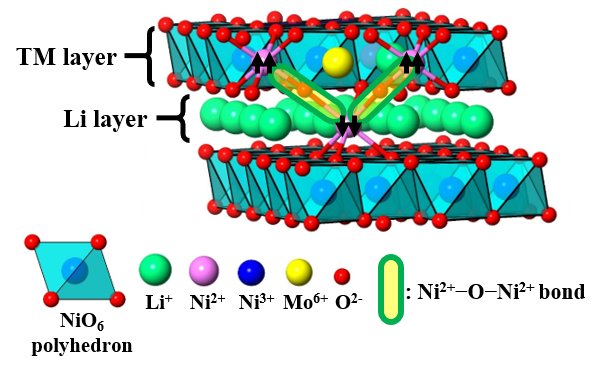


**Supplementary Figure 22:** Li_32_Ni_31_MoO_64_ structure with Li/Ni interlayer mixing. The atomic configuration of Li_32_Ni_31_TaO_64_ is similar to that of Li_32_Ni_31_MoO_64_.

**
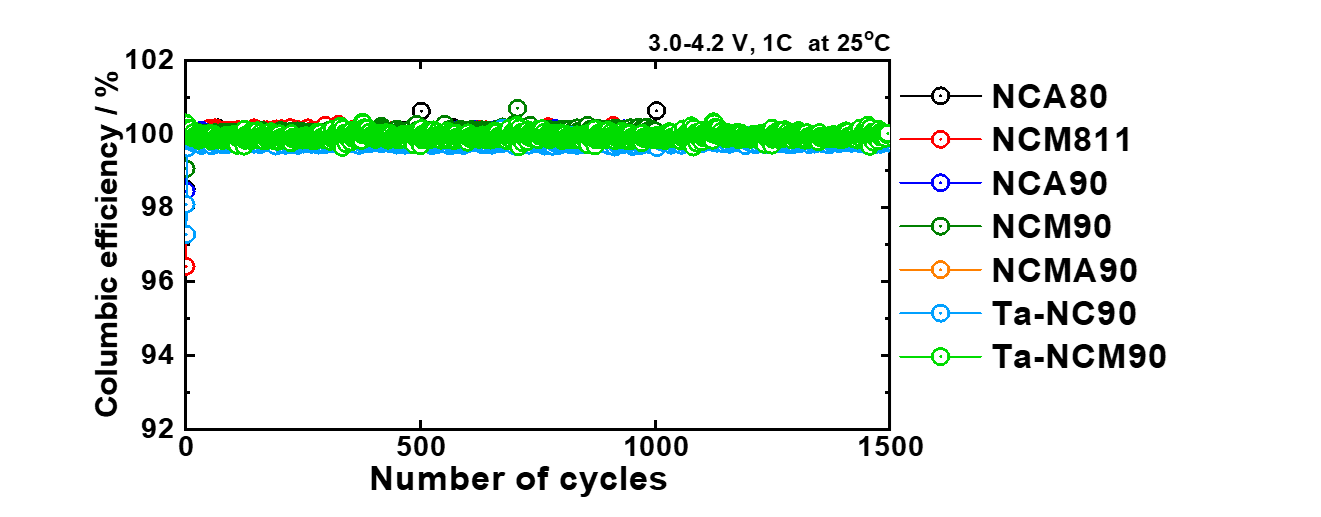
**

**Supplementary Figure 23:** Coulombic efficiencies of NCA80, NCM811, NCA90, NCM90, NCMA90, Ta-NC90, and Ta-NCM90 cathodes in pouch-type full cells.

**
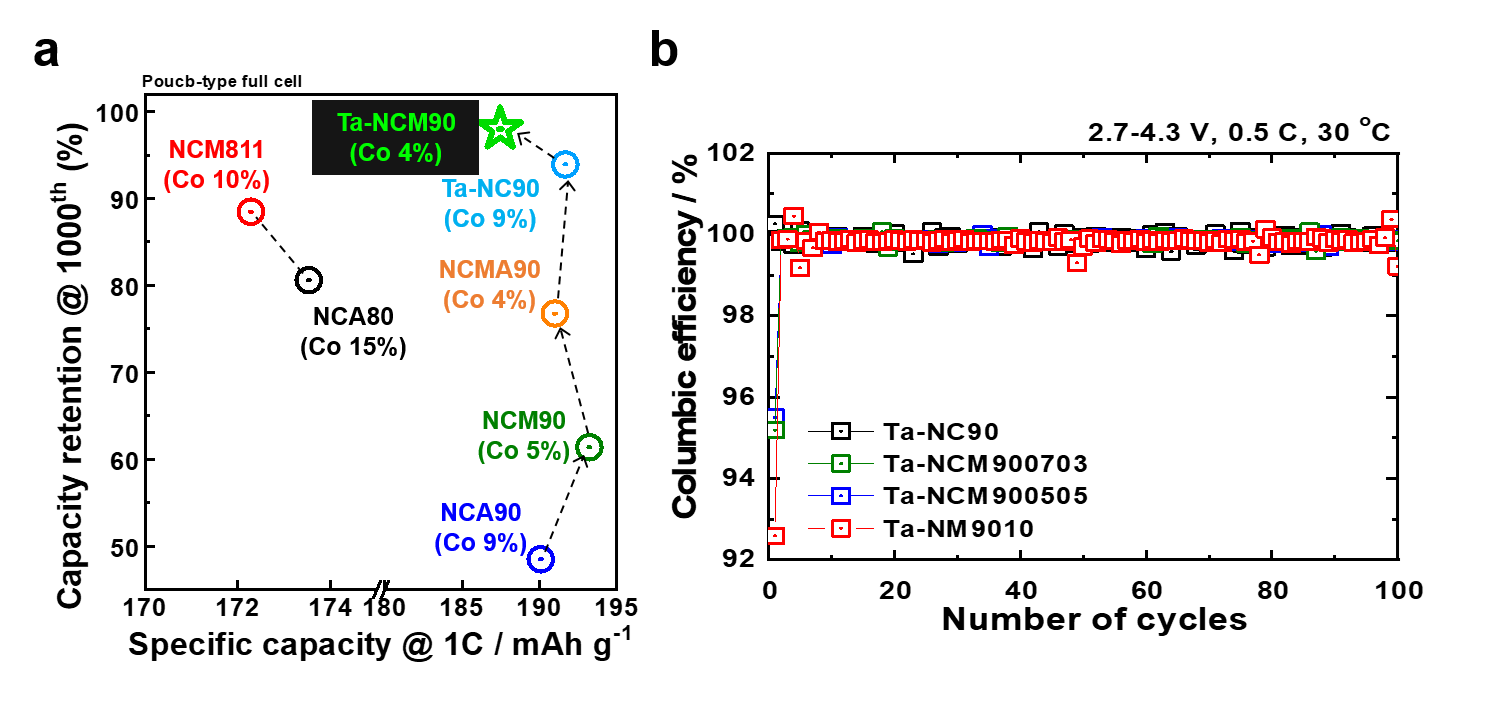
**

**Supplementary Figure 24:** (a) Summary of the relationship between specific capacity, capacity retention, and Co content in Ni-rich cathodes. (b) Coulombic efficiencies of Ta-NC90, Ta-NCM900703, Ta-NCM900505, and Ta-NM9010 cathodes in half cells.

**Supplementary Table 1:** Chemical compositions of NC90, Mg-NC90, Al-NC90, Ti-NC90, Ta-NC90, and Mo-NC90, as determined by ICP-OES. The results indicate successful synthesis of the cathode materials.

| **Chemical composition (atom%)** | | | |
| --- | --- | --- | --- |
| X-NC90  Cathodes | Ni | Co | X = Mg, Al, Ti, Ta, and Mo |
| NC90 | 91.21 | 8.79 | N/A |
| Mg-NC90 | 90.01 | 8.97 | 1.02 |
| Al-NC90 | 89.97 | 8.99 | 1.04 |
| Ti-NC90 | 90.02 | 9.07 | 0.91 |
| Ta-NC90 | 90.041 | 9.01 | 0.95 |
| Mo-NC90 | 89.98 | 9.1 | 0.92 |

**Supplementary Table 2:** Parameters of the fitted regression lines corresponding to Figures 2e–g.

| **Relative primary-particle angle** | |
| --- | --- |
| **Equation** | **y = a + b*x** |
| **Plot** | **B** |
| **Intercept** | **102.23178 ± 2.91751** |
| **Slope** | **−1.26193 ± 0.1053** |
| **R^2^ (COD)** | **0.97954** |
| **Primary-particle aspect ratio** | |
| **Equation** | **y = a + b*x** |
| **Plot** | **B** |
| **Intercept** | **33.68235 ± 5.59984** |
| **Slope** | **9.16688 ± 1.164** |
| **R^2^ (COD)** | **0.95386** |
| **Grain size** | |
| **Equation** | **y = a + b × x** |
| **Plot** | **B** |
| **Intercept** | **117.90995 ± 8.97749** |
| **Slope** | **−97.89878 ± 18.14258** |
| **R^2^ (COD)** | **0.90659** |

**Supplementary Table 3:** Structural characteristics were determined from the Rietveld-refined XRD results of NC90, Mg-NC90, Al-NC90, Ti-NC90, Ta-NC90, and Mo-NC90 cathode powders.

| **Cathodes** | **a_hex_ (Å)** | **c_hex_ (Å)** | **V(Å^3^)** | **Ni^2+^ in**  **Li layer (%)** | **S(R_wp_/R_exp_)** |
| --- | --- | --- | --- | --- | --- |
| NC90  Li[Ni_0.90_Co_0.10_]O_2_ | 2.87051 (2) | 14.17868 (1) | 101.178 (1) | 1.5 | 1.73 |
| Mg-NC90  Li[Ni_0.90_Co_0.09_Mg_0.01_]O_2_ | 2.87034 (3) | 14.18482 (1) | 101.210 (2) | 1.0 | 1.32 |
| Al-NC90  Li[Ni_0.90_Co_0.09_Al_0.01_]O_2_ | 2.86937 (3) | 14.18335 (1) | 101.131 (2) | 1.6 | 1.56 |
| Ti-NC90  Li[Ni_0.90_Co_0.09_Ti_0.01_]O_2_ | 2.87047 (5) | 14.19017 (2) | 101.257 (3) | 1.1 | 1.32 |
| Ta-NC90  Li[Ni_0.90_Co_0.09_Ta_0.01_]O_2_ | 2.87259 (7) | 14.18928 (3) | 101.400 (4) | 2.4 | 1.26 |
| Mo-NC90  Li[Ni_0.90_Co_0.09_Mo_0.01_]O_2_ | 2.87348 (7) | 14.19297 (3) | 101.489 (4) | 3.1 | 1.39 |

**Supplementary Table 4:** Calculated Ni^2+^ and Ni^3+^ areas under the Ni *2p* peaks of the XPS spectra of as-synthesised NC90, Mg-NC90, Al-NC90, Ti-NC90, Ta-NC90, and Mo-NC90 cathode powders.

| **Sample** | **Element** | **Position (eV)** | **Content (%, Area)** |
| --- | --- | --- | --- |
| NC90 | Ni^2+^ | 854.0 | 5.0 |
|  | Ni^3+^ | 855.2 | 95.0 |
| Mg-NC90 | Ni^2+^ | 853.8 | 4.1 |
|  | Ni^3+^ | 855.2 | 95.9 |
| Al-NC90 | Ni^2+^ | 854.0 | 3.5 |
|  | Ni^3+^ | 855.3 | 96.5 |
| Ti-NC90 | Ni^2+^ | 854.1 | 7.0 |
|  | Ni^3+^ | 855.5 | 93.0 |
| Ta-NC90 | Ni^2+^ | 854.3 | 12.4 |
|  | Ni^3+^ | 855.8 | 87.6 |
| Mo-NC90 | Ni^2+^ | 854.3 | 15.0 |
|  | Ni^3+^ | 855.5 | 85.0 |

**Supplementary Table 5:** Computed net moments (μB) of Ni and Ta in Li_32_Ni_32_O_64_ and Li_32_Ni_31_TaO_64_.

| Li_32_Ni_32_O_64_  (no mixing) | | Li_32_Ni_32_O_64_  (mixing) | | Li_32_Ni_31_TaO_64_  (no mixing) | | Li_32_Ni_31_TaO_64_  (mixing) | | |
| --- | --- | --- | --- | --- | --- | --- | --- | --- |
| Atom | Net  moment | Atom | Net  moment | Atom | Net  moment | Atom | Net  moment | |
| Ni1 | 1.094 | Ni1 | **-1.720** | Ni1 | 1.105 | Ni1 | | **-1.713** |
| Ni2 | 1.094 | Ni2 | 1.013 | Ni2 | 1.087 | Ni2 | | 1.082 |
| Ni3 | 1.095 | Ni3 | 1.107 | Ni3 | 1.153 | Ni3 | | 1.082 |
| Ni4 | 1.095 | Ni4 | 1.089 | Ni4 | 1.097 | Ni4 | | 1.087 |
| Ni5 | 1.094 | Ni5 | 1.049 | Ni5 | 1.097 | Ni5 | | 1.097 |
| Ni6 | 1.094 | Ni6 | 1.102 | Ni6 | 1.090 | Ni6 | | 1.076 |
| Ni7 | 1.095 | Ni7 | 1.091 | Ni7 | **1.716** | Ni7 | | **1.718** |
| Ni8 | 1.095 | Ni8 | 1.077 | Ni8 | 1.167 | Ni8 | | 1.089 |
| Ni9 | 1.094 | Ni9 | 1.098 | Ni9 | 1.088 | Ni9 | | 1.107 |
| Ni10 | 1.094 | Ni10 | 1.085 | Ni10 | 1.084 | Ni10 | | 1.091 |
| Ni11 | 1.095 | Ni11 | 1.095 | Ni11 | **1.716** | Ni11 | | **1.712** |
| Ni12 | 1.095 | Ni12 | 1.108 | Ni12 | 1.048 | Ni12 | | 1.094 |
| Ni13 | 1.094 | Ni13 | 1.118 | Ni13 | 1.083 | Ni13 | | 1.171 |
| Ni14 | 1.094 | Ni14 | 1.097 | Ni14 | 1.094 | Ni14 | | 1.066 |
| Ni15 | 1.095 | Ni15 | 1.094 | Ni15 | 1.153 | Ni15 | | 1.100 |
| Ni16 | 1.095 | Ni16 | 1.076 | Ni16 | 1.120 | Ni16 | | 1.079 |
| Ni17 | 1.094 | Ni17 | 1.110 | Ni17 | 1.090 | Ni17 | | 0.989 |
| Ni18 | 1.094 | Ni18 | 1.107 | Ni18 | 1.094 | Ni18 | | 1.072 |
| Ni19 | 1.095 | Ni19 | 1.094 | Ni19 | 1.070 | Ni19 | | 1.090 |
| Ni20 | 1.095 | Ni20 | 1.094 | Ni20 | 1.078 | Ni20 | | 1.091 |
| Ni21 | 1.094 | Ni21 | -1.033 | Ni21 | 1.089 | Ni21 | | 1.094 |
| Ni22 | 1.094 | Ni22 | 1.096 | Ni22 | 1.084 | Ni22 | | 1.086 |
| Ni23 | 1.095 | Ni23 | -1.132 | Ni23 | 1.105 | Ni23 | | 1.096 |
| Ni24 | 1.095 | Ni24 | 1.104 | Ni24 | 1.167 | Ni24 | | 1.075 |
| Ni25 | 1.094 | Ni25 | 1.104 | Ni25 | 1.088 | Ni25 | | 1.000 |
| Ni26 | 1.094 | Ni26 | 1.088 | Ni26 | 1.090 | Ni26 | | 1.084 |
| Ni27 | 1.095 | Ni27 | 1.097 | Ni27 | 1.105 | Ni27 | | 1.108 |
| Ni28 | 1.095 | Ni28 | 1.086 | Ni28 | 1.048 | Ni28 | | 1.144 |
| Ni29 | 1.094 | Ni29 | 1.065 | Ni29 | 1.083 | Ni29 | | 1.088 |
| Ni30 | 1.094 | Ni30 | 1.088 | Ni30 | 1.087 | Ni30 | | 1.089 |
| Ni31 | 1.095 | Ni31 | 1.097 | Ni31 | 1.070 | Ni31 | | 1.082 |
| Ni32 | 1.095 | Ni32 | 1.093 | Ta | **0.000** | Ta | | **0.003** |

**Supplementary Table 6:** Computed net moments (μB) of Ni, Ti, and Mo in Li_32_Ni_31_TiO_64_ and Li_32_Ni_31_MoO_64_.

| Li_32_Ni_31_Ti^4+^O_64_  (mixing) | | Li_32_Ni_31_Mo^6+^O_64_  (mixing) | |
| --- | --- | --- | --- |
| Atom | Net  moment | Atom | Net  moment |
| Ni1 | **1.709** | Ni1 | **-1.714** |
| Ni2 | 1.098 | Ni2 | 1.157 |
| Ni3 | 1.080 | Ni3 | 1.088 |
| Ni4 | 1.092 | Ni4 | 1.091 |
| Ni5 | 1.107 | Ni5 | 1.112 |
| Ni6 | 1.100 | Ni6 | **1.714** |
| Ni7 | 1.191 | Ni7 | **1.690** |
| Ni8 | 1.091 | Ni8 | 1.097 |
| Ni9 | 1.091 | Ni9 | 1.057 |
| Ni10 | 1.084 | Ni10 | 1.086 |
| Ni11 | 1.168 | Ni11 | 1.120 |
| Ni12 | 1.090 | Ni12 | 1.101 |
| Ni13 | 1.146 | Ni13 | 1.054 |
| Ni14 | 1.087 | Ni14 | 1.090 |
| Ni15 | 1.104 | Ni15 | 1.077 |
| Ni16 | 1.077 | Ni16 | 0.942 |
| Ni17 | 1.048 | Ni17 | 1.040 |
| Ni18 | 1.076 | Ni18 | 1.096 |
| Ni19 | 1.085 | Ni19 | 1.100 |
| Ni20 | 1.128 | Ni20 | 1.164 |
| Ni21 | 1.229 | Ni21 | 1.127 |
| Ni22 | 1.107 | Ni22 | 1.098 |
| Ni23 | 1.089 | Ni23 | 1.085 |
| Ni24 | 1.112 | Ni24 | 1.095 |
| Ni25 | 1.231 | Ni25 | **1.717** |
| Ni26 | 1.088 | Ni26 | 1.079 |
| Ni27 | 1.102 | Ni27 | 1.118 |
| Ni28 | 1.124 | Ni28 | 1.090 |
| Ni29 | 1.094 | Ni29 | 1.099 |
| Ni30 | 1.095 | Ni30 | 1.096 |
| Ni31 | 1.101 | Ni31 | 1.127 |
| Ti | **0.013** | Mo | **0.072** |
